# Nucleosome spacing across cell types, diseases, and ages

**DOI:** 10.1093/nar/gkag074

**Published:** 2026-03-05

**Authors:** Milena Bikova, Christopher T Clarkson, Vladimir B Teif

**Affiliations:** School of Life Sciences, University of Essex, Wivenhoe Park, Colchester CO4 3SQ, United Kingdom; School of Life Sciences, University of Essex, Wivenhoe Park, Colchester CO4 3SQ, United Kingdom; School of Life Sciences, University of Essex, Wivenhoe Park, Colchester CO4 3SQ, United Kingdom

## Abstract

Nucleosome spacing patterns in the genome form a unique signature of a given cell, reflecting its chromatin organization and gene expression. Recently, studies of nucleosome spacing have expanded substantially due to the development of novel experimental tools and increased analysis of human samples. This has yielded thousands of high-resolution nucleosome maps across many species and cell types, as well as multiple human datasets that span across different ages and health conditions. With the rapid increase in nucleosome mapping data, their analysis and interpretation have become critically important. Indeed, several discrepancies in nucleosome spacing have been reported recently, using different experimental methods. However, when nucleosome spacing is consistently analysed, it can be linked to biologically important processes: (i) active genomic regions are characterized by shorter distances between nucleosomes in comparison to inactive regions; (ii) cancer cells tend to have shorter distances in comparison to normal cells of the same type; and (iii) ageing usually increases distances between nucleosomes. In many cases, the underlying molecular mechanisms remain to be clarified. Here, we provide a critical analysis of this field, focusing on nucleosome spacing in different types of genomic regions and cell types, as well as changes in cell differentiation, cancer, and ageing.

## Graphical Abstract

**Graphical Abstract:**
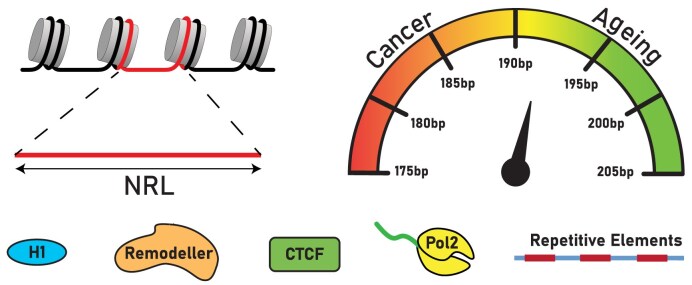

## Introduction

**Figure 1.**
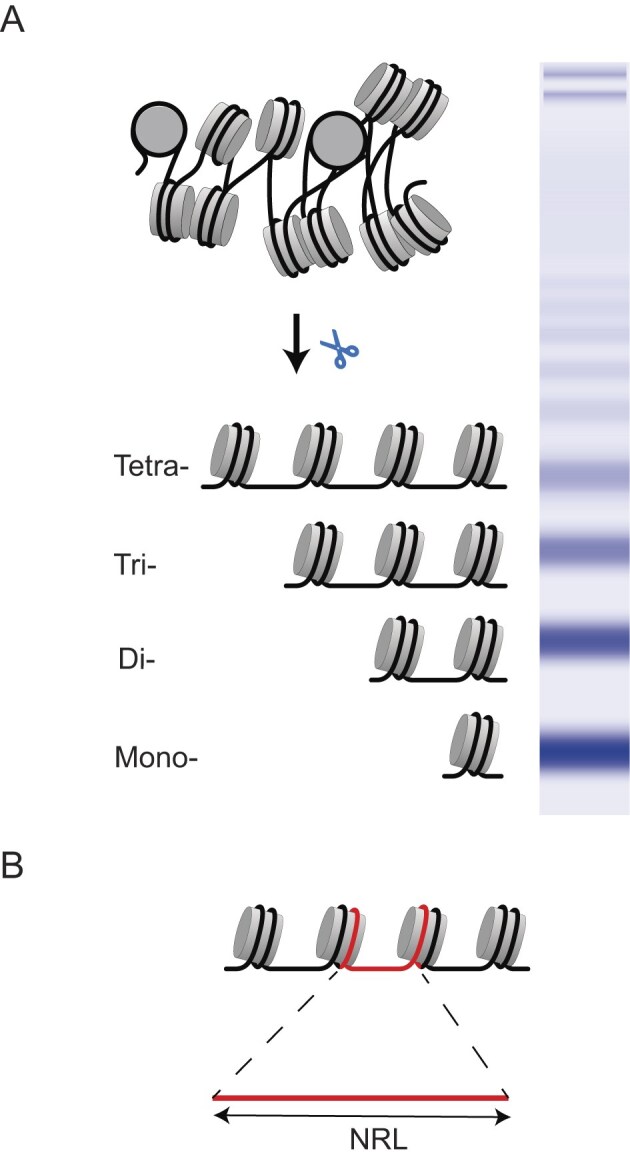
**(A)** Classical gel electrophoresis experiments showing mono-, di-, tri-, tetra-, and further multinucleosome bands upon chromatin digestion. **(B)** The nucleosome repeat length (NRL) is defined as the genomic distance between the centres of two neighbouring nucleosomes.

The human genome is packed in the cell nucleus with the help of about 30 million nucleosomes. The nucleosome consists of ∼147 DNA base pairs (bp) wrapped in 1.65 turns around the histone octamer composed of two copies each of the core histones H2A, H2B, H3, and H4 [1, 2]. Nucleosomes are usually separated by DNA linkers with varying lengths of 10–100 bp. The spacing between nucleosomes reflects many aspects of gene regulation—from current gene expression programs [3–6] to past evolutionary events [7]. This field began in the 1970s with gel electrophoresis images of fragments of DNA isolated from the cytoplasm [8] or from the cell nucleus upon mild chromatin digestion [9–11]. In both cases, one can observe a regular ladder on the gel, with fragment sizes representing multiples of ∼200 base pairs (bp) (Fig. 1A). The explanation of such regular structures required invoking the idea that DNA is packed with a repeating unit of approximately 200 bp [12]. These units were visualised with electron microscopy as the famous beads-on-the-string structures, which were initially called nu-bodies and later became known as nucleosomes [13]. Immediately after the discovery of nucleosomes, scientists started asking whether the distances between nucleosomes change in different cell types, in diseases or during ageing.

In 1989, this field was meticulously summarized in the book “Chromatin” by Kensal van Holde [14]. During the initial period, nucleosome spacing was mostly assessed by estimating the genomic distances between centres of neighbouring nucleosomes [so-called NRL (Fig. 1B)] with the help of classical gel electrophoresis, which allowed only modest resolution. Van Holde included in his book comprehensive tables of NRL values compiled based on dozens of publications but emphasized that it is difficult to interpret what they mean. It is worth noting that at that time, human studies were limited to a small number of immortalized cell lines, and despite large NRL differences across organisms and primary cells, cells of different types grown in culture showed little difference in NRLs [15]—this is an important observation, as detailed in the next sections.

In 2006, Woodcock *et al.* [16] provided an extensive review of the state of the field of nucleosome spacing, suggesting that most dramatic changes in NRL between species and cell types can be attributed to differences in the abundance and composition of variants of linker histones H1. In the 2000s, next-generation sequencing (NGS) techniques allowed mapping nucleosomes with single-bp resolution, which dramatically changed the field [17–19]. By the 2010s, the number of available nucleosome maps increased to a few hundred [20]. In the 2020s, the number of available nucleosome maps increased to tens of thousands [21], allowing more advanced statistical analyses. It is this power of numbers, as well as newer experimental and computational methods, that enabled modern solutions to the classical questions regarding nucleosome spacing in human health and disease. The typical sequencing coverage per sample also increased, allowing the investigation of nucleosome spacing in individual genomic regions, going beyond NRL as a single genome-wide number, towards more localized characteristics of nucleosome spacing. These aspects will be discussed in the next sections.

### MNase-seq and related methods to measure nucleosome spacing

**Figure 2.**
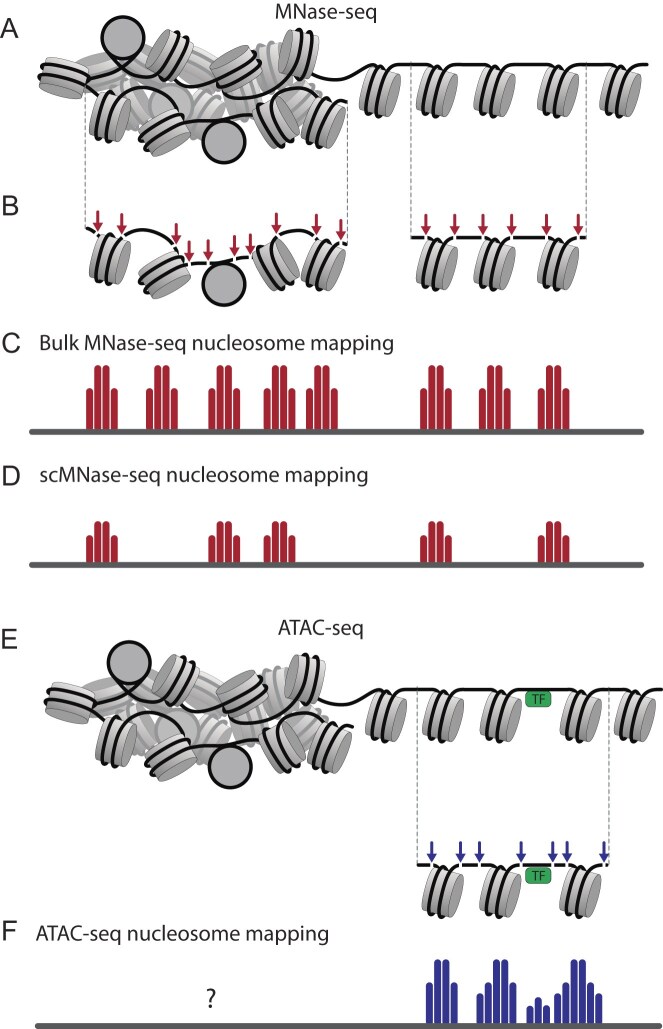
Nucleosome mapping using MNase-seq versus ATAC-seq. **(A)** In MNase-seq, nucleosomes in both open and tightly packed genomic regions are accessible to digestion. MNase preferentially cleaves DNA between nucleosomes and digests DNA until it encounters a histone octamer, which provides a footprint of nucleosome-protected DNA regions. **(B)** Bulk MNase-seq results in averaged maps across millions of cells, effectively capturing all possible nucleosome positioning configurations. **(C)** Single-cell MNase-seq (scMNase-seq) results in a noisier and sparser signal. The resulting footprints still represent nucleosome-protected regions, but not all nucleosomes are represented. **(D)** In ATAC-seq, open regions can be accessed by the enzyme Tn5 transposase, which can insert primers in regions free from the binding of nucleosomes and transcription factors (TFs). **(E)** For open chromatin regions, nucleosome maps can be obtained from ATAC-seq similar to MNase-seq. **(F)** Closed, tightly packed chromatin regions may be less represented in ATAC-seq nucleosome maps.

Since the advent of NGS, many methods have been developed to measure nucleosome positioning and spacing [3–6, 22]. Two decades after its introduction, MNase-seq (Micrococcal Nuclease digestion followed by sequencing) remains the most widely used technique for nucleosome positioning [17–19]. It is based on the ability of micrococcal nuclease (MNase) to cleave spacer DNA in chromatin and then digest it until an obstacle, such as the nucleosome, is encountered. Thus, MNase digestion followed by sequencing allows one to map genomic regions protected by nucleosomes (Fig. 2A). MNase has known sequence preferences for DNA cleavage [23, 24], which may lead to large [25] or small biases in MNase-seq [26], depending most notably on GC-content. Furthermore, this may be amplified by different representations of MNase-resistant (“stable”) vs. -sensitive (so-called “fragile”) nucleosomes [27], as well as nucleosome breathing/unwrapping/sliding [28], leading to changed nucleosome maps depending on the level of digestion [29, 30]. Despite these limitations, MNase-seq is widely used and can be considered a quantitative technique [31].

The classical MNase-seq method described above is typically applied to a population containing 10^4^–10^6^ cells in the case of mammalian systems. As with other sequencing methods, MNase-seq also exists in a single-cell version, called scMNase-seq [32, 33]. Unlike bulk MNase-seq, where a large ensemble of molecules allows even rare cleavage events to be represented in the final signal (Fig. 2B and C), in scMNase-seq many nucleosomes can be lost from mapping because MNase did not make its random cut near them (Fig. 2D). Thus, the single-cell MNase-seq signal is significantly sparser and is challenging to apply to nucleosome spacing analysis.

Several MNase-seq-related methods have also been developed, such as a combination of MNase with exonuclease III or other endonucleases that help to digest DNA more precisely at the nucleosome borders [34], as well as MNase-assisted ChIP-seq with an antibody against histone H3 (MNase-H3-ChIP-seq) [35, 36]. The latter method has been shown to give NRL values consistent with MNase-seq [36].

### Additional experimental methods to study nucleosome spacing

Many alternative methods to map nucleosomes have been developed. One group of complementary methods is based on the idea of cutting DNA without MNase, e.g. using directed chemical cleavage at certain histone locations [37–42]. For example, a method based on H4 mutant H4S47C to cleave DNA near the nucleosome dyad by hydroxyl radicals [40] showed differences from MNase-seq at transcription starts and CTCF binding sites, although NRL calculations with this method were generally consistent with MNase-seq [43, 44].

In recent years, another method of mapping chromatin accessibility, ATAC-seq, has become increasingly popular [45]. The number of publications with the keyword “ATAC-seq” currently surpasses that of “MNase-seq” by about 10-fold, and the prevalence of ATAC-seq is even larger for human datasets. ATAC-seq is based on the use of a hyperactive form of the enzyme Tn5 transposase, which cleaves accessible DNA and inserts sequencing adaptors. Thus, ATAC-seq identifies regions free from protection by DNA-bound transcription factors (TFs) and nucleosomes (Fig. 2E and F). Tn5 transposase can insert adaptors close to the nucleosome entry/exit, as well as close to the middle of the linker DNA between nucleosomes [46], with the sizes of the majority of ATAC-seq DNA fragments corresponding to 50–100 bp regions between nucleosomes, and the size of the next most abundant fraction reflecting the size of nucleosomal DNA plus linker. It is worth keeping in mind that unlike MNase-seq, which profiles both open and closed chromatin regions, ATAC-seq mostly profiles open chromatin near bound TFs, and many regions located away from bound TFs may be out of the picture.

From the onset of the use of ATAC-seq, it has been suggested that it is suitable for the study of nucleosome spacing. In fact, a major software package for ATAC-seq analysis, NucleoATAC [47], calculates the median distance between nucleosome dyads. However, the median nucleosome dyad-dyad distances calculated in several studies using this software are around 250–270 bp [48–50], significantly larger than NRLs determined with MNase-seq for these systems. On the other hand, our re-calculation of NRLs from raw ATAC-seq data using the algorithms described in the next section gives NRLs of ∼190 bp, compatible with classical nucleosome positioning studies (see Supplementary Fig. S1 and the methodological description in Supplementary Materials). This computational issue might have contributed to the delay of the adoption of ATAC-seq for nucleosome spacing analysis. Recent works showed that ATAC-seq can be effectively used to determine NRL changes between conditions [51], but it requires appropriate computational analyses, as detailed in the next section.

Another promising class of experimental approaches to measure nucleosome spacing is based on mapping genomic DNA–DNA contacts, which can also be used to derive nucleosome positions. These methods include radiation-induced spatially correlated cleavage of DNA with sequencing (RICC-seq) [52], and variations of chromatin contact mapping with single-nucleosome resolution such as Micro-C [53, 54]. A recently introduced method based on a proximity-tagging approach utilizing a diverse array of nucleic acid tags, called Proximity Copy Paste (PCP), can also generate high-resolution maps of nucleosome–nucleosome contacts [55]. The advantage of such methods is that they can provide distances not only between next-neighbour nucleosomes, but also between second-, third-neighbours, etc. This is particularly useful when assessing the 3D structure of nucleosome clutches in different genomic regions [44, 56–60] and other aspects of the relationship between nucleosome positioning and higher-order genome folding [6, 61–67, 68]. 3D-inspired resolution of nucleosome spacing also aligns well with long-read sequencing techniques such as Nanopore [69–71] or PacBio [72, 73], which allow one to obtain information about nucleosome spacing in a single molecule using the fact that the length of DNA fragments produced by long-read sequencing corresponds to different numbers of nucleosomes on the same section of the chromatin fiber (so-called Fiber-seq). Single-fiber nucleosome arrangement can also be investigated using methyltransferases to map DNA methylation and nucleosome locations simultaneously [74, 75]. Such methods can provide important information about the interplay of methylation and nucleosome positioning on the same molecule, although the resolution is limited by the distance between neighbouring CpGs. When single-bp resolution is needed, MNase-based methods remain the primary choice for nucleosome mapping.

**Figure 3.**
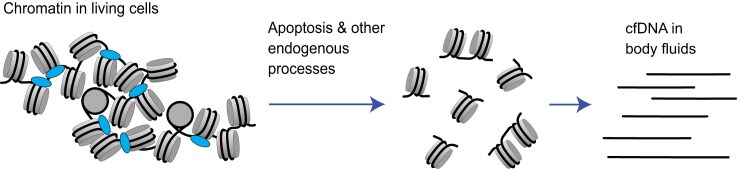
cfDNA released from cells into bodily fluids as part of endogenous processes such as apoptosis reflects genomic regions protected from digestion in chromatin, mostly appearing in mononucleosome sizes, as well as stretches of a few nucleosomes.

Finally, a new and developing field of studying cell-free DNA (cfDNA) allows one to analyse nucleosome spacing based on the distributions of nucleosome-protected genomic regions derived from short pieces of circulating DNA in bodily fluids [21, 76] (Fig. 3). Such pieces of cfDNA originate from different types of cells (mostly from white blood cells for a healthy person), in processes such as apoptosis, where the genome of a dying cell is digested by endogenous nucleases, preferentially between nucleosomes, in analogy to MNase-seq experiments [77]. It is worth keeping in mind that endogenous mammalian nucleases have different properties from MNase, which may contribute to differences between cfDNA-based nucleosome maps and MNase-seq [78].

### NRL calculations from NGS data

There are several approaches to calculate NRL from NGS data. These approaches are generally consistent with each other, but do not converge to the same values.

**Figure 4.**
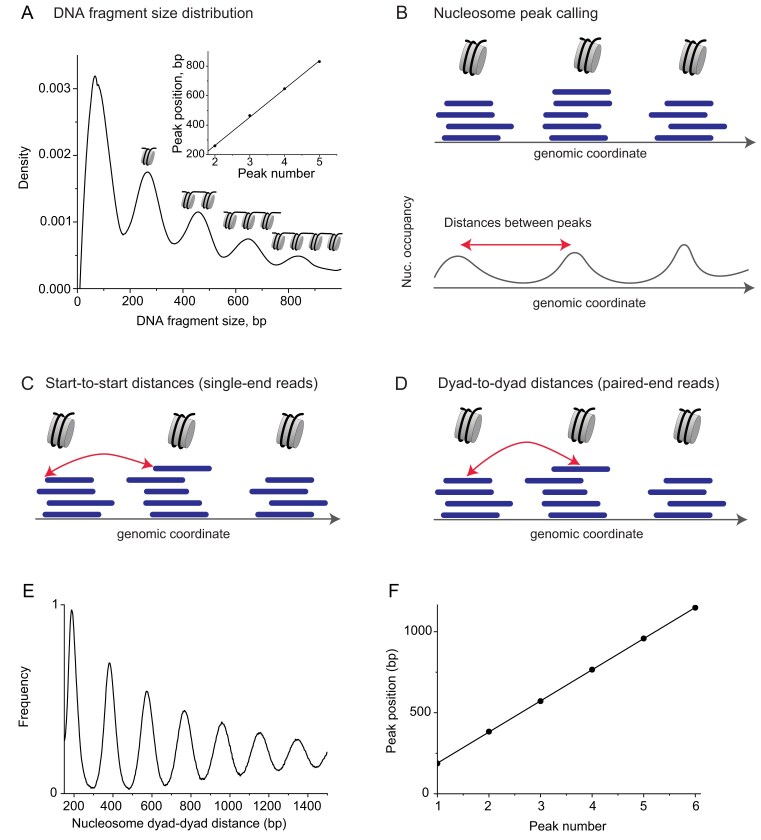
Different strategies for NRL calculation from NGS data. **(A)** Calculating the frequency distribution of fragment sizes and then performing linear regression through the summits of the peaks of this distribution. This approach relies on the abundance of multinucleosome fragments of partially digested chromatin. This method is applicable to many types of data; the current example shows data for ATAC-seq. **(B)** Calculating the nucleosome occupancy profile, calling nucleosome occupancy peaks, and measuring distances between peaks—this approach is applicable to all nucleosome mapping techniques, but risks missing ill-defined nucleosome peaks. (**C, D**) Calculating distances between individual DNA reads (fragments protected by mono-nucleosomes) based on MNase-seq or similar methods. **(C)** In the case of single-end sequencing, only the coordinates of the starts of nucleosomes are known. **(D)** In the case of paired-end sequencing, the coordinates of both ends of the nucleosome are known, which allows one to calculate the locations of nucleosome dyads (midpoints of nucleosomal DNA fragments). **(E)** Calculation of the frequency of distances between nucleosome dyads defined as in panel (D). **(F)** Calculation of NRL as a linear regression of locations of the peak summits of the distribution of nucleosome distances from panel (D). The slope of the linear fit gives the NRL.

a) *Using DNA fragment size distribution*. The first approach to calculate NRL is based on the analogy to classical gel electrophoresis experiments (Fig. 4A). In this method, the summits of the peaks of the fragment size distribution corresponding to mono-, di-, tri-nucleosomes, and further multinucleosome complexes, are collected and used in linear regression to determine NRL as the slope of the linear fit across these points. This approach works particularly well with MNase-seq based on long-read sequencing (e.g. Nanopore), which retains fragments longer than mononucleosomes [69, 70], as well as ATAC-seq (Fig. 4A and Supplementary Fig. S1). Our recent work showed that short-read sequencing of cfDNA also generates a substantial amount of multinucleosome fragments, which allow one to determine NRL using this type of analysis [79].

b) *Using peak calling of nucleosome locations*. The power of NGS is that NRL can be defined genome-wide, for a subset of genomic regions, or locally over a single region. In the latter case, it may be possible to define the genomic nucleosome locations using peak calling, and once the locations of nucleosome occupancy peaks are resolved, the NRL can be calculated as the average of distances between summits of neighbouring peaks (Fig. 4B). A similar approach is possible when calculating NRL for a subset of regions that can be aligned with respect to some anchor points, e.g. transcription start sites (TSSs) or TF-binding sites, in which case the nucleosome occupancy landscape represents an aggregated profile averaged across all such genomic regions. However, in the case of larger genomic regions or for the whole-genome analysis, state of the art peak calling methods applied to mammalian nucleosome occupancy data can identify dyad locations for only a fraction of nucleosomes due to the inherent fuzziness of nucleosome organization [80]. Consequently, calculations based on such incomplete nucleosome maps may lead to incorrect estimation of NRLs.

c) *Using autocorrelation functions of nucleosome starts*. A more stringent method of NRL calculation from NGS data uses the genomic coordinates of the original sequenced DNA fragments (raw data) without calculating nucleosome occupancy and avoiding peak calling steps. Historically, MNase-seq applications first used single-end sequencing, and NRL calculation based on DNA fragments was performed with so-called phasograms (or autocorrelation functions in mathematical terminology) of distances between genomic coordinates of DNA fragment starts [81] (Fig. 4C). This way, one does not have knowledge of the exact locations of nucleosome dyads and must assume that the start of the DNA fragment is close to the nucleosome entry.

d) *Using autocorrelation functions of nucleosome dyads*. When paired-end sequencing became widespread for MNase-seq it allowed determining nucleosome dyad locations as the midpoint between the start and the end coordinates of each sequenced DNA fragment [82] (Fig. 4D). This allows using the autocorrelation function for dyad-dyad distances as in the previous method. Since the nucleosome dyad is better defined than nucleosome start/end, this method allows more precise determination of NRL using linear regression (Figs. 4E and F), with less than 1bp error even for moderate sequencing coverage of ∼200 million paired-end reads for the human genome [36].

The choice of one of these methods for NRL calculation depends on the available experimental data and biological questions, but it is important to use the same method consistently. Our analysis indicates that when properly implemented, the DNA fragment size distribution methods (Fig. 4A) and autocorrelation methods (Fig. 4C–F) give similar results. The DNA fragment size distribution typically has a higher error of NRL definition, but it may be preferred for ultra-low-coverage datasets and non-NGS DNA fragment size-based methods, where the calculation of the autocorrelation function is not possible. In our experience, the method based on peak calling of individual nucleosome locations (Fig. 4B) should be avoided unless it is applied to a subset of small well-defined regions such as TSSs. As mentioned above, mammalian nucleosome organization is inherently very fuzzy and therefore the identification of stable non-overlapping positions of all nucleosomes from peak calling is conceptually not possible, while the use of peak calling-based incomplete nucleosome maps results in a calculation of NRLs incompatible with other methods. This issue is not limited to ATAC-seq [48–50] (Supplementary Fig. S1), but is also characteristic of some studies dealing with cfDNA [83]. In such cases, our recommendation is to use methods (a), (c), or (d) instead.

### Molecular mechanisms affecting nucleosome spacing

**Figure 5.**
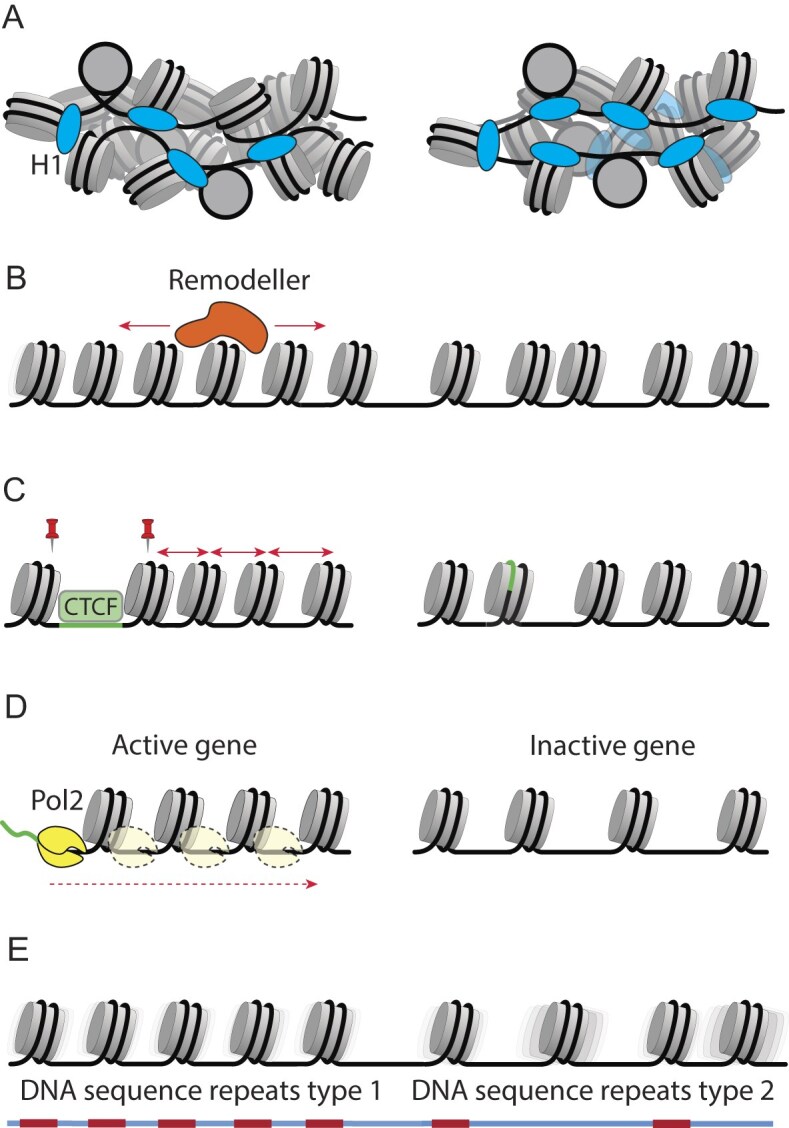
Molecular mechanisms affecting nucleosome spacing. **(A)** Linker histones H1 and nonhistone chromatin proteins which compete with H1s and modulate nucleosome spacing through structural and electrostatic mechanisms. **(B)** Chromatin remodellers actively reposition nucleosomes following context-dependent rules. **(C)** Cell state-dependent chromatin boundaries formed by CTCF and other structural proteins, as well as associated recruitment of chromatin remodellers which space nucleosomes. **(D)** Gene activity associated with remodeller action and RNA polymerases transcribing through the nucleosomes, leading to smaller distances between nucleosomes in regulatory regions and gene bodies. **(E)** DNA sequence repeats of different types.

The main molecular mechanisms affecting nucleosome spacing can be summarized as follows (Fig. 5):

a. *Linker histones*. Linker histones H1 and their analogues exert global effects on NRL, which are particularly pronounced when comparing different organisms and cell types [84], as we will detail in the next sections. In experimental systems, manipulations of linker histone abundance lead to significant NRL changes [51, 85, 86]. Linker histones can potentially affect nucleosome spacing through changes in the chromatin electrostatics [87–89], as well as by exerting pressure by binding and unbinding stochastically between nucleosomes [90, 91], or through bridging interactions, as they can stabilise some nucleosome–nucleosome conformations [92]. Interestingly, H1 binding itself is influenced by linker DNA length and trajectory, which in turn affects NRLs [93]. A related class of molecular mechanisms of nucleosome spacing involves non-histone chromatin proteins that bind in competition or in cooperation with linker histones, e.g. methyl-binding proteins such as MeCP2 [94] and high-mobility group proteins such as HMGB1 [95].

b. *Chromatin remodellers*. Nucleosome remodellers are ATP-dependent molecular motors, which reposition nucleosomes and as such exert major effects on nucleosome spacing. They can do this through several mechanisms, which include the “measurement” of the flanking DNA length [96], applying a “ruler” element to measure the distance between nucleosomes [97], sometimes recognizing dinucleosomes [98] and even sensing the density of nucleosomes to modulate nucleosome spacing [68]. Different remodeller classes have distinct rules of nucleosome repositioning [99, 100], which may be context- and species-specific. For example, CHD1 is one of the major players in nucleosome spacing in yeast [101], where genes remodelled by CHD1 have short spacing, while those remodelled by ISW1 and ISW2 have longer spacing [102]. Studies in mouse cells suggested that the action of the remodeller SNF2H (SMARCA5) is associated with the smallest NRL near bound CTCF proteins [43, 103], consistent with the general role of this remodeller in nucleosome spacing [104]. CHD4 has a role in the asymmetry of nucleosome arrays near the architectural protein CTCF, which is involved in 3D genome organization [43], while SMARCA5 is continuously required for the maintenance of this nucleosome spacing [105]. Interestingly, some cancer-associated mutations in core histones that interfere with remodeller recognition can change nucleosome spacing, e.g. through ISWI-mediated nucleosome sliding [106].

c. *Boundary effects*. Nucleosome positioning is strongly affected by boundaries that create arrays of nucleosomes adjacent to their positions. Boundaries can be formed by DNA sequences with extremely high or low affinity for the histone octamer or binding sites of proteins that exclude nucleosomes [107, 108]. As a result, nucleosome spacing depends on the differential locations of such boundaries, for example those defined by differential binding of CTCF [43, 109, 110]. While the existence of a boundary pre-defines the location of a phased nucleosome array, the distances between nucleosomes in such an array may be further modulated. For example, CTCF-dependent nucleosome spacing is regulated by chromatin remodellers, some of which are mentioned above, and is particularly important in the context of cell differentiation and cell cycle progression [105, 111–113]. CTCF represents an important specific case, since ordered nucleosome arrays near its binding sites account for up to 10% of the human or mouse genome [43]. However, several other DNA-binding proteins may also act as strong boundaries and recruit remodellers, creating local differences in nucleosome spacing [43]. For example, PU.1 is one of the TFs beyond CTCF that can form phased nucleosome arrays [114].

d. *Transcriptional effects*. Transcription by RNA polymerase II is a major reorganizer of nucleosome spacing, both via the act of transcription itself as well as by the associated transcriptional machinery, which includes TFs, remodellers, histone modification enzymes, and histone chaperones [6, 115, 116]. Changes in nucleosome spacing in genic regions represent a mixture of several effects. These include the boundary effect of the TSS, which is usually nucleosome-depleted, combined with the effect of chromatin remodellers that are essential to establish the nucleosome array next to the TSS [117]. RNA polymerase can transcribe through the nucleosome *in vivo* [118, 119], which plays a role in the establishment of nucleosome spacing and regularity inside the gene. Exact mechanisms of the latter effect on NRL are not clear, but it is known that genes with higher transcriptional activity have shorter NRL [69, 81] with fuzzier (less regular) nucleosome arrays [69, 120]. Changes in histone modifications or histone variants linked to transcription activation or repression, enzymes that modify them and proteins that recognise these, can also affect nucleosome spacing. For example, depletion of histone 3 lysine 4 (H3K4) demethylase KDM5B results in changes of nucleosome spacing at TSS [121]. The incorporation of histone variant H2A.Z instead of canonical H2A may also result in different structures of nucleosome fibers [122].

c. *DNA sequence effects*. Finally, nucleosome spacing is affected by DNA sequence [123, 124], including DNA sequence repeats as well as nucleosome-favouring and nucleosome-excluding motifs. The examples of the former include telomeres [125], alpha-satellite repeats [126], and ALU repeats [127], as well as any other repeats with sizes comparable to NRL [91, 128, 129]. The examples of the latter include arrays of so-called strong nucleosomes [130], and any regularities arising from nucleosome-excluding sequences such as Poly(dA:dT) tracts [131]. DNA sequence repeats are hard-wired in the genome, but nucleosome spacing associated with them can change because of epigenetic regulation. This can be, e.g. through a change in DNA methylation or histone modifications affecting the 3D packing of nucleosome arrays in certain fractions of these repeats [132].

### Interplay of nucleosome spacing and 3D genome organization

Differences in nucleosome spacing can affect 3D genome organization in several ways, which has been extensively reviewed elsewhere [6, 61–65, 67, 68, 133, 134]. One aspect noticed in the 1970s is that, due to the 10-bp repeat of the DNA double helix, there may be a preference for the sizes of DNA linkers between nucleosomes to be quantized with 10-bp steps, as observed, e.g. when treated with exonuclease III [135]. Such quantization is less pronounced in MNase-seq experiments, which typically show smooth dyad-dyad distance distributions as in Fig. 4E, but becomes more visible in chemical nucleosome mapping [40]. However, even in the latter case, the genome-average linker size calculated across the whole distribution may not reflect multiples of ten. For example, consider a distribution with two equal major peaks at 10n and 10(n + 1) bp. The average of such a distribution can be located closer to 10n + 5 bp. When two neighbouring nucleosomes are separated by distances of 10n bp, they are in the same rotational register. In this case, the cylindrical shapes of nucleosomes (a nucleosome can be represented with a ∼11 nm wide disc as a foundation of a ∼6 nm tall cylinder) are arranged such that their flat faces (the disks) are stacked vertically on top of each other. In contrast, when the distances follow some other pattern, e.g. 10n + 5 bp, which is another frequently reported mode of quantization, the flat faces of neighbouring nucleosome cylinders are roughly parallel, and nucleosomes are packed side-by-side (e.g. reviewed in [133]). Thus, switching between 10n and 10n + 5 bp linker quantization offers interesting biological effects for the topology of nucleosome arrays [63, 136, 137]. However, nucleosome organization in mammalian chromatin is typically more diverse, and the polymorphism of chromatin fibers can be modulated by variations in local NRLs [138, 139]. In the next sections we will talk about NRL changes as small as 1–2 bp, or a few base pairs, that do not follow clear quantization patterns, in the context of ageing and cancer.

Importantly, *in vitro* reconstruction of chromatin fibers showed that NRL values have large effects on 3D nucleosome organization [140]. In the latter experiments, an NRL of 197 bp allowed the formation of the 30-nm fibers, whereas an NRL of 167 bp was too short to allow the flexibility needed for compact packing. Multiple experimental and modelling studies have been conducted since then (reviewed in [133]). The current understanding is that apart from local regions where nucleosome arrangement is better defined, forming so-called clutches or nanodomains [44, 56–60], mammalian genomes have liquid-like, fiber-less organization of nucleosome arrays [141]. Local fluctuations of linker histone concentrations and other chromatin proteins can further fine-tune and diversify nucleosome packing [142, 143]. The concentration of nucleosomes *per se* has a smaller effect on chromatin packing/accessibility than NRL [66]. At the intermediate scale, genomic domains with preferred nucleosome spacing are associated with 3D-defined compartments of different types. Such domains have been shown to have NRL-dependent 3D packing *in silico* [144], *in vitro* [97], and *in vivo* [145–147], and can have distinct biological properties such as changed DNA damage repair efficiency [148]. Potential mechanisms that can account for such NRL-associated 3D compartmentalization depend on electrostatic interactions [147] and can include so-called structural homology, where nucleosome arrays with similarly spaced nucleosomes will preferentially interact with each other [149, 150], an effect which may be particularly pronounced in genomic regions where nucleosome positioning is affected by homotypic clustering of DNA sequence repeats [151].

### NRL differences across species

Multiple observations going back to the pre-NGS era revealed large NRL variations in different species [14]. Such differences go beyond simply having a different genomic sequence, as demonstrated by studies where the host organism has overwritten the rules of nucleosome positioning for foreign sequences inserted into it [152]. One of the major sources of inter-species NRL variation is the relative abundance of core and linker histones [84, 86], with varying effects of histone subtypes [85]. Significant NRL differences can be observed even within yeast species [152]. The shortest NRL of 154 bp has been reported for *Schizosaccharomyces pombe*, which lacks linker histones [153, 154]. A different yeast species, *Saccharomyces cerevisiae*, where linker histone Hho1p is essential for chromatin compaction in the stationary phase [155], has a larger NRL of 167 bp. Note that both of these NRL values were determined using the algorithm from Fig. 4B looking at nucleosome locations near TSS [154]. Since it is a TSS-specific analysis, it may also depend on different transcription levels in these species. Furthermore, these two yeast species have different chromatin remodellers, which may provide another explanation [102, 156].

Two other classical examples of inter-species variability in NRL are chicken erythrocytes and sea urchin sperm. Unlike human erythrocytes, which do not have a nucleus, chicken erythrocytes have nuclei containing nucleosomes separated by linker histones H1 (the same as mammals) and in addition linker histones H5 (a bird-specific subtype). In chicken erythrocytes, H1 and H5 together account for about 1.3 linker histone molecules per nucleosome [157], which is significantly larger than in mammalian cells [16]. This leads to an NRL around 212 bp in mature chicken erythrocytes [158, 159]. During chicken erythropoiesis, when the content of linker histone H5 changes, NRL values change in the range between 205 and 218 bp, following the amount of H5 present [160]. The largest NRL ever reported is for sperm of sea urchins, which reaches 240 bp [161]. Unlike human sperm, where most nucleosomes are replaced by polyamines, sea urchin sperm retains the beads-on-a-string nucleosomal structure, but with different histone variants. Its testes-specific linker histones are bulkier and carry a larger positive charge in comparison to their somatic counterparts [162]. Therefore, when somatic linker histones are replaced by testis-specific variants, the NRL in sea urchin cells increases from 218 bp for gastrula cells to 240 bp in sperm [161]. Note that even 218 bp is significantly above typical NRLs in mammalian systems. Thus, different species exhibit a large variability in genome-wide NRLs, and it remains to be determined how critical it is. Furthermore, such variability can also be observed in different genomic regions of the same cell, where it has functional consequences. In the next section we will focus on the latter effect.

### NRL in different types of genomic regions

Even within the same cell type in the same organism, different types of genomic regions are characterized by different nucleosome spacing [see Table 1 with our NRL calculations for human lymphoblastoid cells and Supplementary Fig. S2 for mouse embryonic stem cells (ESCs)]. Some genomic regions have hard-wired nucleosome spacing due to their repetitive structure. In humans, telomere repeats contain tightly packed nucleosomes with NRLs as small as 157 bp, whereas *in vitro* assembly at telomeric repeats results in ∼130 bp nucleosome arrays [163]. Centromeric alpha-satellite repeats have an NRL of 171 bp [126], and a subset of ALU repeats defines an NRL of 167 bp [127]. When the size of the DNA sequence repeat does not match the size of the chromatosome (nucleosome plus linker), then the NRL is established by the interplay between boundary conditions set by DNA sequences and non-sequence factors such as concentrations of different proteins [91, 128, 164]. Other types of genomic regions have variable NRLs depending on their activity, enrichment with epigenetic factors and 3D compaction. For coding regions, the higher the gene expression, the smaller the NRL wihtin the corresponding gene body and associated *cis-*regulatory modules [36, 40, 69, 81, 165]. For example, our analysis shows that in human lymphoblastoid cells, promoter-proximal genome regions of actively transcribed genes have an NRL of ∼179 bp, weakly transcribed genes have an NRL of ∼186 bp, while inactive/poised promoters have an NRL of ∼197 bp (Table 1). The effects of transcription on NRL are likely causative, with RNA polymerase rearranging nucleosomes as it passes through them [115], resulting in more ordered arrays [6, 69], and chromatin remodellers recruited to rearrange nucleosomes as a function of transcriptional activity. Some histone modifications associated with active/inactive regions can directly modulate the chromatin fiber structure or recruit remodellers, which affects NRL in the corresponding chromatin states [68, 82, 166, 167]. Regions surrounding TF-binding sites also have smaller than genome-average NRL, provided that nucleosome–nucleosome distances are measured on one side of the TF [43]. A particularly interesting case is the 3D demarcating protein CTCF, which organizes regular arrays of ∼10 nucleosomes in its vicinity [109]. The NRL in such arrays is anticorrelated with the strength of CTCF binding, decreasing with both the increase of the height of the experimental CTCF ChIP-seq peak and predicted CTCF motif strength [43]. On the other hand, heterochromatin in cultured human and mouse cells typically has a larger NRL around 190 bp [44, 168], which is close to the genome average. However, different types of heterochromatin have different organization and as a result different NRLs [44]. When chromatin states change with time, NRL can change as well [43, 44]. One practical consequence of the NRL variability across the genome is that experimental methods that enrich for certain genomic features may return significantly different averaged NRLs.

**Table 1.** NRL values in different types of genomic regions calculated based on the experimental dataset of MNase-seq in lymphoblastoid cells from seven people reported by Gaffney *et al.* [169] using the computational methodology demonstrated in Fig. 4D–F for ChromHMM-defined chromatin states [170] (detailed in Supplementary Materials)

Feature	State	NRL, bp	StDev, bp
Promoters	Active	176.5	±1.9
	Weak	181.3	±1.9
	Inactive/poised	192.7	±1.8
Genes	Highly transcribed	190.3	±1.8
	Weakly transcribed	193.5	±1.9
Heterochromatin	Polycomb-repressed	194	±1.5
	Low epigenomic signal	192.9	±1.3

### Nucleosome spacing in cell differentiation

There are many cases of NRL changes in cell differentiation. One well-known example comes from brain development. Pre-NGS studies in a number of mammalian organisms including rabbit [171], ox [172] and rat [173, 174] found that cerebral cortex neurons have shorter NRL (160–170 bp), while glial cells have longer NRL (180–200 bp). Recently, this was also confirmed in mice using MNase-seq [175]. The latter study compared mouse dorsal root ganglia neurons (NRL ∼165 bp), cortical oligodendrocyte precursor cells (NRL ∼182 bp) and cortical astrocytes (NRL ∼183 bp). During organism development, NRL in cortical neurons decreases from 200 bp before birth to 170 bp at 14 days [176].

An interesting recent example of NRL changes during development was reported in a study in *Plasmodium falciparum*, which is the malaria-causing parasite [177]. The authors of this study calculated NRLs from MNase-seq data at eight time points of its developmental cycle and showed that NRL changes as a smooth, continuous function, decreasing from 185 bp 5 h post-invasion down to 176 bp 30 h post-invasion, and then increasing again to ∼182 bp 40 h post-invasion. Notably, this microorganism does not have H1 linker histones.

Another example of NRL change during development comes from the recent studies of rod photoreceptors [178], which found that during mouse rod maturation, NRL increases from ∼190 to ∼206 bp, while immature retina cells contain a larger fraction of nucleosomes with short linkers [179, 180]. Both effects of NRL changes during the development of cortical neurons and rod photoreceptors are believed to be driven by changes in chromatin composition varying from around one H1 molecule per nucleosome to around 0.5 H1 molecules per nucleosome in cortical neurons [16] and 1.3 H1 molecules per nucleosome in rod photoreceptors [178]. It is worth noting that these levels of H1 and associated NRLs may be subject to heterogeneity and may change in specific genomic regions.

Experimental depletion of linker histone variants in mouse ESCs indeed caused an NRL decrease from ∼190 bp in wild type ESCs to ∼174 bp in triple-H1 null ESCs [86]. A recent study performed in murine CD8^+^ T cells depleted of three subtypes of H1 also showed NRL reduction by about 10 bp [51]. It should be noted that in cortical neurons, a smaller abundance of H1 is also associated with a high presence of MeCP2 (methyl CpG binding protein 2) [181]. Since MeCP2 is known to compete with H1 for DNA binding [94], MeCP2 is another potential candidate for the explanation of NRL change during neuron differentiation, although recent tests of this hypothesis have been inconclusive [182]. MeCP2 and H1 can also mediate liquid–liquid phase separation (LLPS) of chromatin [183], which may have an effect on NRL, but this relationship is yet to be explored.

On the other hand, in cases where there are no large changes in the content of linker histones, differentiation is usually associated with modest NRL changes associated with gene activity. For example, MNase-seq based NRLs in mouse ESCs range from 186 bp [82] to 189 bp [44], while neural progenitor cells (NPCs) have NRL of 193 bp [82]. Other types of mouse cells such as thymus and liver have slightly larger NRLs around 196 bp [184] (gel electrophoresis-based estimate). Stem cell chromatin in general is believed to be more open than the chromatin of differentiated cells because it is more transcriptionally active [185]. A recent study has reevaluated this conclusion using mild chromatin digestion with DFF/CAD (DNA Fragmentation Factor/Caspase-Activated DNase) followed by gel electrophoresis, reporting NRLs as large as 200–206 bp for both stem cells and differentiated cells [186]. The discrepancy between DFF/CAD gel electrophoresis and MNase-seq may arise from milder digestion by DFF/CAD, thereby enriching for a subset of multinucleosome fragments corresponding to nucleosome clutches that are more difficult to digest. Such digestion-resistant nanodomains with compact chromatin can have longer linkers, which can bend more easily to satisfy topological and steric constraints [138, 140, 179, 187–189]. This effect is related to NRL changes associated with gene activity in different genomic regions described in the previous section—more active genomic regions have smaller NRLs (Table 1 and Supplementary Fig. S1). In general, as cells undergo differentiation, their chromatin nanodomains can appear/disappear, merge and rearrange [44, 179], resulting in changes in NRL both locally and globally.

Thus, cell differentiation is associated with a large variability of NRLs, partly reflecting the specialization of cells (cells of a given type have a specified nuclear morphology) and partly reflecting differences in transcriptional activity as well as the redistribution of chromatin compaction states. When organisms age or acquire diseases, their phenotype may change further, as discussed in the next sections.

### Nucleosome spacing in ageing

Early investigations in ageing mice and rats conducted in the pre-NGS era have reported NRL differences of up to 10 bp, mostly in the context of different brain cells [174, 176, 190–192]. However, due to the timeline of rodent development these may represent a mixture of effects of cell differentiation and ageing. Pre-NGS era studies in general had low experimental resolution, which needs to be taken into account when interpreting the reports that did not detect NRL changes in lymphocytes [193] and human skin fibroblasts [194] from people of different ages. Such studies were also statistically underpowered, since age-associated effects of 1–2 bp NRL change detailed below require significantly larger cohorts to be detectable.

NGS-era studies have clearly demonstrated the existence of ageing-associated chromatin defects which precede DNA damage accumulation in human fibroblasts [195], but until recently there was no simple answer to the question of systematic changes in nucleosome spacing with age. Studies in yeast [196] and Drosophila [197] showed that ageing is associated with a loss of histones, with an estimated 50% decrease of overall nucleosome occupancy in ageing yeast [198]. Studies in mice concluded that the total histone H3 level is not significantly affected in ageing, although some genomic regions increased or decreased H3 presence [199]. A more recent study performed using MNase-seq reported overall decreased nucleosome density with age [200]. It is worth noting that since nucleosome spacing is defined by exact locations of two neighbouring nucleosomes, a simple change of core histone density, e.g. as accompanies healthy ageing [196–198, 200], does not automatically mean a change in NRL, unless the ratio of core/linker histones changes. A recent study of nucleosome dyad distances in neutrophils of young and old mice provided a valuable ATAC-seq dataset [48], which can be analysed with appropriate methods (see above and Supplementary Fig. S1 regarding reanalysis of ATAC-seq based NRLs). In our recent publication, we investigated the effect of ageing on nucleosome spacing using sequenced cfDNA from > 100 people of different ages [79]. This study allowed us to conclude that NRL tends to increase with age, and we have even constructed an ageing clock predicting age from nucleosome positioning with ∼3–4 years precision [79]. This provided the answer to the long-standing question—NRL does increase with age, at least for NRL in white blood cells, which are responsible for the majority of cfDNA in blood plasma. Other cell types may age differently. For example, atomic force microscopy of hyper-quiescent chromatin state formed during aging in murine liver suggested that in this chromatin fraction NRL decreases in aged hepatocytes [201]. Another recent study performed in human skin fibroblasts showed that ageing decreases the number of CENP-A/CENP-C proteins associated with centromeres [202]. Since human centromeric chromatin is enriched with 171-bp satellite repeats, this may potentially affect nucleosome spacing in the centromeric fraction of nucleosomes. Thus, in the future it would be interesting to expand NGS studies of nucleosome spacing in ageing to different types of cells and organisms.

### Nucleosome spacing in cancer

The investigations of NRL in cancer started soon after the discovery of the nucleosome, and in fact most human cell lines used in early NRL studies were established from cancer patients [14]. However, the latter fact also introduced a confounding factor, because it appeared that most cell lines grown in media, independently of their source, were characterized by similar NRL values around 180–190 bp [15]. Cell lines may be characterized by higher proliferation in comparison with their primary cell counterparts, and since active chromatin is associated with shorter NRLs, this may have confounded the analyses based on cell lines. For example, our previous analysis of differentiation of the human myeloid leukemia cell line HL-60 found similar NRL values around 192 bp for undifferentiated and differentiated HL-60 cells [168]. On the other hand, primary B-cells purified from the peripheral blood of healthy human volunteers have NRLs around 201 bp [35]. Our recent study showed that B-cells from human patients with chronic lymphocytic leukaemia (CLL) have a decreased NRL, with this decrease being less pronounced in less aggressive CLL (∼198 bp) and more pronounced in more aggressive CLL subtypes (∼196 bp) [35].

The decrease in NRL in B-cells from CLL patients mentioned above is in line with our recent study, which reported a 7–10 bp NRL decrease in breast tissues from breast cancer patients [36]. In the latter case, direct comparisons were made using paired breast tissues taken from the tumour and the adjacent breast area from the same patients. This publication described potential mechanisms for the observed NRL shortening: differential binding of linker histones (H1X and H1.4 having the largest effects), changes in DNA methylation, DNA sequence repeats status and gene expression. By comparing cancer-specific nucleosome spacing in tissues and cfDNA, this work suggested the possible use of nucleosome spacing analysis in cfDNA-based liquid biopsies for patient diagnostics. Importantly, even in cancer patients, cfDNA mostly comes from white blood cells rather than from tumour tissues, and thus it may reflect cancer effects indirectly (e.g. through immune response) rather than through changes that happen in tumour tissues. It is worth noting that NRL changes in tumour tissues in breast cancer patients were not limited to promoters or active genes and were observed in different types of genomic regions. In fact, repetitive genomic regions had the largest NRL changes [36]. This suggests that additional factors such as GC content and DNA sequence repeats state/accessibility (which may be influenced e.g. by DNA methylation) may play a role in the local NRL changes [36]. This publication was followed by more recent studies that applied this approach to analyse NRL changes in cfDNA from cancer patients, focusing on differences between active and inactive chromatin regions [203, 204]. As discussed above, NRL changes can reflect global differences in chromatin compaction/accessibility, and shorter NRL is typically associated with more active/less compact chromatin (Table 1). In this respect, it is interesting to note another recent report that cancer cells may have globally increased accessibility in comparison to their normal counterparts [205].

While the amount of linker histones usually does not change dramatically in ageing or cancer, their composition/genomic distribution can change. Notably, mutations in linker histones are known to be among the most frequent drivers of blood cancers, constituting 30%–50% in different types of lymphomas [206], with about 11% of all tumours harbouring somatic histone mutations [207]. Thus, even if the amounts of linker histones are not significantly altered in cancer, their ability to interact with DNA may be altered through mutations, providing a mechanism affecting nucleosome spacing. Considering large variations in NRL during neuronal development mentioned above, H1 mutations may account for chromatin changes not only in blood cancers, but also in neurodegenerative diseases [208].

Overall, despite significant recent progress, our understanding of the molecular mechanisms of the changes in nucleosome spacing in cancer is far from complete. On a practical side, the whole nucleosome spacing landscape is changed in cancer, not just a single integral parameter such as NRL, which allows distinguishing between normal and cancer samples [35, 36]. Clinically, such nucleosomics analyses can be carried out by reconstructing the nucleosome positioning landscapes from cfDNA extracted from body fluids, e.g. blood plasma, which makes them a promising, minimally invasive diagnostic test [21, 76, 77].

### Summary and outlook

**Figure 6.**
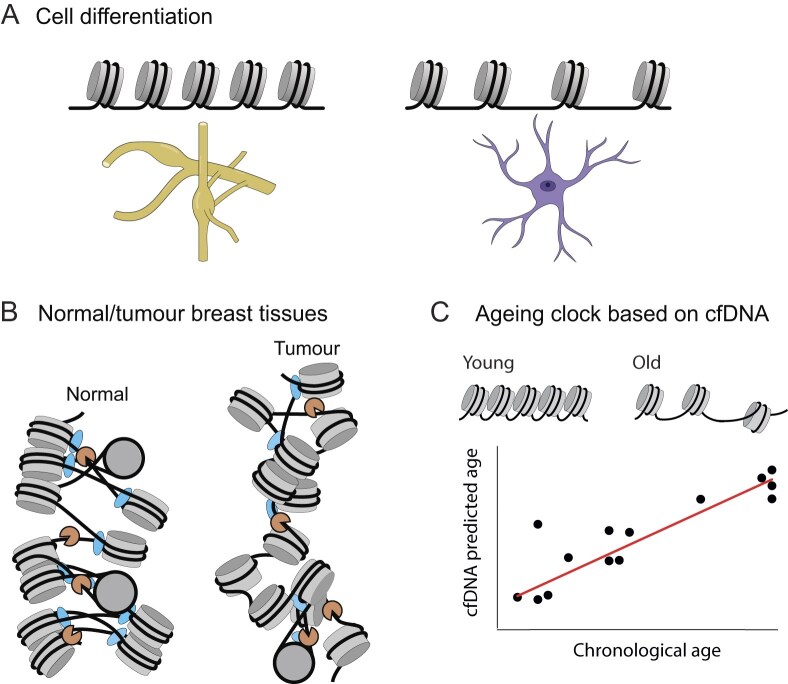
Examples of NRL changes in biological systems. **(A)** Cell differentiation leads to NRL changes between different cell types, e.g. mouse dorsal root ganglia neurons (NRL ∼165 bp) versus cortical astrocytes (NRL ∼183 bp) [175]. Schematic cell shapes are adapted from an image created in BioRender (https://BioRender.com/89trj2t). **(B)** Paired normal versus tumour breast tissues show NRL shortening in cancer (figure adapted from [36] under the CC BY 4.0 licence (https://creativecommons.org/licenses/by/4.0/)). **(C)** Nucleosome positioning derived from cfDNA of human volunteers shows NRL increase with age (figure reprinted from [79] under the CC BY 4.0 licence (https://creativecommons.org/licenses/by/4.0/)).

The accumulation of a large number of high-resolution nucleosome maps allows us to investigate nucleosome spacing landscapes systematically, across the genome and between different species, cell types and cell transitions. Genome-wide NRL changes across species may be mostly due to the differences in the composition/abundance of linker histones and nonhistone chromatin proteins. Local nucleosome spacing changes within the same cell are mostly associated with DNA sequence and context-specific action of chromatin remodellers and gene activity (Fig. 5). Nucleosome spacing differences between cell types and cell transitions such as ageing and cancer may involve all of the above mechanisms, as well as cell type/transition-specific changes of DNA and histone modifications, chromatin boundaries and accessibility/activity of DNA sequence repeats (Fig. 6). The detailed molecular mechanisms of NRL changes in cell differentiation, ageing and cancer in many cases require further clarification. Recent developments allowing the prediction of age based on nucleosome positioning derived from cfDNA [79], as well as diagnosing/stratifying cancer patients based on nucleosome positioning [35] are quite promising. The importance of the nucleosome spacing research is further growing considering the applications for liquid biopsies based on cfDNA [36, 76, 79]. All of this requires further investigation of the molecular aspects of local and global NRL changes. Given that we now have the experimental and computational tools for determining nucleosome spacing in local genomic regions with single-bp resolution for a single person or condition, this exciting area will likely keep us busy in the future.

## Supplementary Material

gkag074_Supplemental_File

## Data Availability

Published datasets used for the analysis are available in the Gene Expression Omnibus database under accession numbers GSE36979 and GSE82127, and in the Short Read Archive (SRR11700292).

## Notes

Present address: Department of Neuromuscular Diseases, Institute of Neurology, University College London, London WC1N 3BG, United Kingdom
