# Supplementary material for: Nucleosome spacing across cell types, diseases, and ages
Source: Nucleic Acids Res. 2026 Mar 5;54(5):gkag074. doi: 10.1093/nar/gkag074 (PMC12961432; doi:10.1093/nar/gkag074)
Supplement: gkag074_Supplemental_File [file gkag074_supplemental_file.pdf]

Supplementary Materials

Nucleosome spacing across cell types, diseases and ages

Milena Bikova[1], Christopher T. Clarkson[1,2], Vladimir B. Teif[1,]*

[1] School of Life Sciences, University of Essex, CO4 3SQ, Wivenhoe Park, Colchester, UK

[2] University College London, Gower St, Bloomsbury, London WC1E 6BT, UK

*To whom correspondence should be addressed. E-mail: vteif@essex.ac.uk

Calculation of NRL based on ATAC-seq

As mentioned in the main text, the NRL determined from ATAC-seq with the NucleoATAC software (1), reported across several publications, is outside of the range of typical values based on MNase-seq. To clarify this issue, we have used as an example ATAC-seq data from Lu et al, 2021 (2). Lu et al studied ageing in mice, and reported NRL values around 260-270 bp, which they calculated with NucleoATAC. In the example below, we are using the raw data from one of their samples downloaded from the Short Read Archive (SRR11700292), which we have mapped with Bowtie2 to the mouse reference genome mm9. Supplementary Figure S1A shows the DNA fragment size distribution for this sample, smoothed with a 50-bp, rank 2, Savitzky-Golay filter. We have determined the summits of all peaks and performed linear regression on these data points (Supplementary Figure S1B), which resulted in NRL = 191 bp. The latter value is within the typical range of values determined for such cells with MNase-seq. This suggests, that the issue with the large NRL values determined based on ATAC-seq by NucleoATAC is not experimental, but computational.

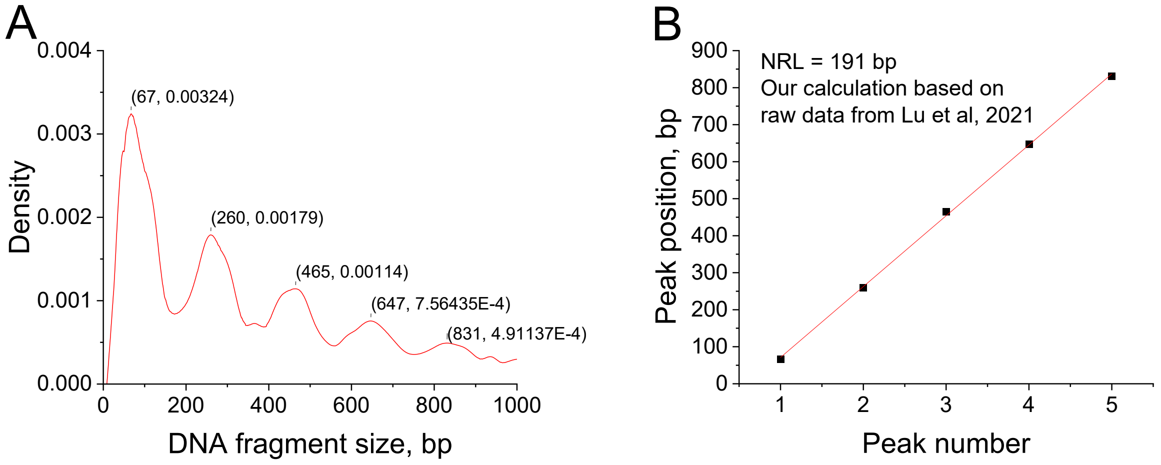

Figure S1. A) DNA fragment size distribution for sample SRR11700292 from Lu et al, 2021 (2). The data is smoothed with a 50-bp, rank 2, Savitzky-Golay filter. The numbers on the graph indicate the locations of the peak summits. B) Linear regression using the summits of five peaks from panel (A) results in NRL = 191 bp. Note that the first peak corresponds to DNA fragments from nucleosome-free regions. Excluding peak 1 and taking into account only peaks 2, 3, 4, 5 results in a similar linear fit, with NRL = 190 bp.

Calculation of NRLs included in Table1.

The NRL values reported in Table 1 were derived from the experimental dataset of Gaffney et al (3) (GSE36979) using the computational methodology demonstrated in Figure 4D for ChromHMM-defined chromatin states (4). The coordinates of genomic regions associated with 15 ChromHMM states in the human genome assembly hg19 were obtained from the UCSC Genome Browser track wgEncodeBroadHmmGm12878HMM.bed and expanded by 2000 base pairs on each side with BedTools Slop (5). Paired-end MNase-seq reads reported by Gaffney et al for lymphoblastoid cells from seven healthy people (3) were size-selected to include only fragment sizes between 120 and 180 bp and analysed separately within each ChromHMM-defined type of genomic regions. NRL calculations were then performed as described previously (6,7) using NucTools (8) to calculate the distributions of nucleosome dyad-dyad distances from paired-end reads. Regions with pile ups of more than five reads with the same dyad coordinate were discarded. This was followed by the linear regression of the summits of the dyad-dyad distribution with NRLcalc (9). The original ChromHMM notation contained some states with less clearly defined biological meaning in terms of types of genomic features – these states were not included in Table 1. The following states included in Table 1 were renamed for clarity:

“Genes” / “highly transcribed” – state 10 “transcriptional elongation” in the ChromHMM track;

“DNA repeats” / “enriched with all marks” – state 15 “Repetitive/CNV” in the ChromHMM track.

Previously we have conducted similar analysis for MNase-seq in mouse embryonic stem cells (ESCs) (10) (GSE82127) based on the reported 15-state ChromHMM annotation reported for ESCs (11) (included in the PhD dissertation of Christopher T Clarkson, University of Essex, 2019). Supplementary Figure S1 reports these NRL values for ChromHMM states in ESCs.

State	H3K36me3	H3K4me1	H3K27ac	Pol2	Input	H3K4me3	CTCF	H3K27me3	Coverage (Mean)	Length (Mean)		NRL	# nucs
1	81	1	1	4	1	0	3	3	4.2	2.5	Transcription Elongation	171.07 +/- 3.2	20698127
2	16	1	0	1	1	0	1	2	6.7	2.7	Weakly Transcribed	182.48 +/- 5.2	33079165
3	84	60	33	12	1	1	6	7	0.8	0.9	Transcriptional Transition	161.44 +/- 8.2	3839024
4	90	57	62	34	2	88	12	13	0.6	0.7	Weak/poised Enhancer	161.21 +/- 1.2	776935
5	5	23	7	8	1	74	4	6	0.4	0.5	Active Promoter	177.59 +/- 4.2	4248064
6	10	89	70	62	5	96	36	41	0.2	0.6	Strong Enhancer	165.87 +/- 5.2	1734829
7	4	11	85	61	6	97	23	20	0.4	0.9	Active Promoter	179.55 +/- 2.2	4544288
8	6	62	81	15	2	3	9	8	0.7	0.7	Strong Enhancer	177.59 +/- 5.2	4477105
9	3	37	4	5	1	0	2	6	2.1	0.8	Weak/poised Enhancer	176.61 +/- 2.2	13476133
10	6	54	7	17	3	53	17	89	0.3	0.9	Poised Promoter	172.7 +/- 0.2	2263842
11	2	2	0	1	1	0	2	49	1.3	1.8	Repressed	186.06 +/- 2.2	15171168
12	1	0	0	0	0	0	0	6	18.5	17.7	Heterochromatin	188.02 +/- 1.2	253474334
13	0	0	0	0	0	0	0	1	44.1	120.7	Heterochromatin	187.04 +/- 2.2	34897408
14	0	1	0	2	1	0	1	2	19.7	5.7	Heterochromatin	174.33 +/- 2.2	23629370
15	4	12	3	21	2	1	41	12	0.7	0.4	Insulator	169.12 +/- 6.2	2468568
	Chromatin mark observation frequency (%)								(%)	(Kb)			

Figure S2. NRL values in different types of genomic regions calculated based on the experimental dataset of Voong et al (MNase-seq in mouse embryonic stem cells (ESCs) (10)) using the reported 15-state ChromHMM annotation reported for ESCs (11). Note that this analysis has larger error values in comparison with Table 1 due to lower sequencing coverage of the dataset of Voong et al in comparison with the dataset of Gaffney et al.

Supplementary References

1. Schep, A.N., Buenrostro, J.D., Denny, S.K., Schwartz, K., Sherlock, G. and Greenleaf, W.J. (2015) Structured nucleosome fingerprints enable high-resolution mapping of chromatin architecture within regulatory regions. *Genome Research*, **25**, 1757-1770.
2. Lu, R.J., Taylor, S., Contrepois, K., Kim, M., Bravo, J.I., Ellenberger, M., Sampathkumar, N.K. and Benayoun, B.A. (2021) Multi-omic profiling of primary mouse neutrophils predicts a pattern of sex and age-related functional regulation. *Nat Aging*, **1**, 715-733.
3. Gaffney, D.J., McVicker, G., Pai, A.A., Fondufe-Mittendorf, Y.N., Lewellen, N., Michelini, K., Widom, J., Gilad, Y. and Pritchard, J.K. (2012) Controls of nucleosome positioning in the human genome. *PLoS Genet*, **8**, e1003036.
4. Ernst, J. and Kellis, M. (2012) ChromHMM: automating chromatin-state discovery and characterization. *Nat Methods*, **9**, 215-216.
5. Quinlan, A.R. (2014) BEDTools: The Swiss-Army Tool for Genome Feature Analysis. *Curr Protoc Bioinformatics*, **47**, 11 12 11-34.
6. Jacob, D.R., Guiblet, W.M., Mamayusupova, H., Shtumpf, M., Ciuta, I., Ruje, L., Gretton, S., Bikova, M., Correa, C., Dellow, E. *et al.* (2024) Nucleosome reorganisation in breast cancer tissues. *Clin Epigenetics*, **16**, 50.
7. Shtumpf, M., Jeong, S., Bikova, M., Mamayusupova, H., Ruje, L. and Teif, V.B. (2024) Aging clock based on nucleosome reorganisation derived from cell-free DNA. *Aging cell*, e14100.
8. Vainshtein, Y., Rippe, K. and Teif, V.B. (2017) NucTools: analysis of chromatin feature occupancy profiles from high-throughput sequencing data. *BMC Genomics*, **18**, 158.
9. Clarkson, C.T., Deeks, E.A., Samarista, R., Mamayusupova, H., Zhurkin, V.B. and Teif, V.B. (2019) CTCF-dependent chromatin boundaries formed by asymmetric nucleosome arrays with decreased linker length. *Nucleic Acids Res*, **47**, 11181-11196.
10. Voong, L.N., Xi, L., Sebeson, A.C., Xiong, B., Wang, J.P. and Wang, X. (2016) Insights into Nucleosome Organization in Mouse Embryonic Stem Cells through Chemical Mapping. *Cell*, **167**, 1555-1570.
11. Bogu, G.K., Vizan, P., Stanton, L.W., Beato, M., Di Croce, L. and Marti-Renom, M.A. (2015) Chromatin and RNA Maps Reveal Regulatory Long Noncoding RNAs in Mouse. *Mol Cell Biol*, **36**, 809-819.
